# Supplementary material for: Structure Elucidation of In Situ Generated Chiral Hypervalent Iodine Complexes via Vibrational Circular Dichroism (VCD)
Source: Angew Chem Int Ed Engl. 2022 Jun 21;61(50):e202204624. doi: 10.1002/anie.202204624 (PMC10084129; doi:10.1002/anie.202204624)
Supplement: Supplementary file 1 — Supporting Information [file ANIE-61-0-s001.pdf]

Angewandte Chemie
Eine Zeitschrift der Gesellschaft Deutscher Chemiker

Supporting Information

Structure Elucidation of *In Situ* Generated Chiral Hypervalent Iodine Complexes via Vibrational Circular Dichroism (VCD)

T. P. Golub, A. H. Abazid, B. J. Nachtsheim, C. Merten**

DOI: 10.1002/anie.202204624

Communication

Received: 29.März 2022

Structure Elucidation of In Situ Generated Chiral Hypervalent Iodine Complexes via Vibrational Circular Dichroism (VCD)

Tino^^P. Golub,[a] Dr. Ayham^^H. Abazid,[b] Prof.^^Dr. Boris^^J. Nachtsheim,*[b] Prof.^^Dr. Christian Merten0000-0002-4106-1905*[a]

[a] <orgName/>Ruhr Universität Bochum, <orgDiv/>Fakultät für Chemie und Biochemie, Organische Chemie II
<street/>Universitätsstraße 150, <postCode/>44801 <city/>Bochum (<country/>Germany)
E-mail: christian.merten@ruhr-uni-bochum.de
<urla>https://www.mertenlab.de</url>

[b] <orgName/>Universität Bremen, <orgDiv/>Fachbereich 2----Biologie und Chemie, Institut für Organische und Analytische Chemie (<country/>Germany)
E-mail: nachtsheim@uni-bremen.de

Due to their instability and reactivity, the structure of in situ generated iodane(III) species is challenging to characterize. VCD spectroscopy reveals a cationic species as the most important intermediate of a chiral triazole-containing aryl-λ^3-iodane catalyst under oxidative conditions.

The structure of in situ generated chiral aryl-λ^3-iodanes obtained under oxidative reaction conditions was not yet observable with experimental techniques and their proposed structures are purely based on DFT calculations. Herein, we establish vibrational circular dichroism (VCD) spectroscopy as an applicable experimental technique to verify DFT-calculated chiral iodane structures. Based on a chiral triazole-substituted iodoarene catalyst, we were able to elucidate a yet undescribed cationic chiral iodane as the most populated intermediate under oxidative conditions with a significant intermolecular N<C->I-interaction and no significant interactions with tosylate or *m*-chlorobenzoic acid as potential anionic ligands. Instead, aggregation of these substrates was found, which resulted in the formation of a non-coordinating anionic hydrogen bonded complex. The importance of VCD as a crucial experimental observable is further highlighted by the fact that our initial structural proposal, that was purely based on DFT calculations, could be falsified.

Asymmetric Catalysis
Computational Chemistry

Hypervalent Iodine
Stereochemistry
Vibrational Spectroscopy

@MertenLab (on the figure, please link: @ChemieBiochemie and @SolvationSci)

Chiral iodoarenes are the most versatile organocatalytic oxidation catalysts applicable in a plethora of enantioselective umpolung reactions.[1] The in situ generation of chiral hypervalent iodine compounds, in particular aryl-λ^3-iodanes, through oxidation of chiral monovalent iodoarene precursors with a terminal co-oxidant is crucial in iodoarene-catalyzed reactions since the resulting hypervalent iodine(III) complexes are the key reactive intermediates that orchestrate the stereoselective C<C->X-coupling.[2] Due to their superior reactivity, these oxidized aryl-λ^3-iodanes are hard to isolate and hence important structural insides of these in situ oxidized reactive species remain largely unexplored.

Even after 25^^years of intensive developments in chiral iodane catalysis and a great number of available chiral iodoarene catalysts,[3] structural information of these compounds in their oxidized form is still scarce. Wirth and co-workers proposed donor-stabilized cationic iodosyl species stabilized by a tethered methyl ether based on DFT calculations (Scheme^^1<schr1>A).[4] Similar pseudocyclic cationic hydroxy(tosyloxy)iodo compounds were also described based on solid state structures and DFT-calculations.[5] A solid-state structure was observed for a derivative of the widely applied C_2-symmetric resorcinol lactates developed by Uyanik and Ishihara, which features hydrogen bonding interactions between amide NH-protons and iodane-bound alkoxylates (Scheme^^1<xschr1>B).[6] Another solid-state structure was observed by Wirth and co-workers through oxidation of a chiral iodotetralone, in which no significant interactions of tethered ether oxygen donors was observed.[7] Our group recently established the chiral triazole-substituted iodoarene catalyst **1** and proposed the in situ generated iodane **2-OTs** as the most stabile species based on DFT calculations. In this initial estimate the triazole acts as a hydrogen bond acceptor that stabilizes a strongly twisted hypervalent bond.

Although these diverse proposed structures give an initial estimate about the potential secondary interactions between internal and external ligands and the chiral hypervalent iodine center, ligand exchange processes in solution are likely to result in significant structural

rearrangements. Structure elucidation of in situ generated reactive hypervalent iodine(III) compounds in solution is still challenging and usually such information is based on DFT calculations exclusively.[8] This is mainly due to the fact that common tools such as NMR spectroscopy can give at most indirect information about the coordination sphere around the iodine and consequently, information on the preferred conformation of the catalyst cannot be reliably derived. So far, mechanistic studies rely solely on computed structures of intermediates and reaction pathways without having a direct link to an experimental observable. Hence, reliable spectroscopic methods for the structure elucidation of in situ generated chiral iodanes in their operational reactive form in a realistic oxidation media are highly desirable and would allow a rational design of chiral iodoarene catalysts and significantly widen their scope of applications in enantioselective oxidative coupling chemistry.

Taking the chiral triazole-substituted iodoarene shown in Scheme^^1<xschr1>C as representative example,[9] we herein establish vibrational circular dichroism (VCD) spectroscopy in combination with density functional theory (DFT) calculations as a powerful tool to elucidate the structure of these mostly unknown reactive intermediates under real oxidative reaction conditions. VCD spectroscopy is the chiral version of infrared spectroscopy[10] and frequently exploited for the determination of absolute configurations.[11] In contrast to electronic circular dichroism (ECD), that is recorded for electronic transitions in the UV/Vis region, it does not require any dedicated chromophores as all chiral molecules are IR-active. It is therefore generally applicable for structure elucidation of chiral molecules in solution. The analysis of a VCD spectrum is based on the comparison with computed spectra and thereby provides a link between an experimental observable and computed structural preferences. In fact, detailed knowledge on conformational preferences is a key requirement for a VCD analysis since VCD signatures of individual conformers of a chiral molecule are distinctly different, while their corresponding IR spectra are similar. Consequently, VCD spectra analysis fails when important conformers are not considered in the calculations. In several case studies, we demonstrated that the unique conformational sensitivity of VCD spectroscopy can help gaining novel insights into the structural preferences of asymmetric catalysts.[12] For the example of an ion-pair based catalyst,[13] we demonstrated that a chiral

phosphate anion, that is not involved in the catalytic cycle itself, imposes chiral conformational preferences onto an achiral Mn^{III}-salen cation.[14] For chiral thiourea catalysts, we utilized VCD spectroscopy to reveal their binding mode and the relative orientation of catalyst and substrate in hydrogen-bonded complexes.[15] Jørgensen--Hayashi-type prolinol ethers[16] and MacMillian’s imidazolidone catalysts were investigated as examples for covalent organocatalysts.[17]

These applications of VCD spectroscopy made us envision that the conformational preference of chiral iodine(III) species should also be characterizable. A particular challenge anticipated for such study arises from the fact that the active iodine(III) species must be generated in situ and that it cannot be isolated from the side products of the activation reaction. For the model system,[9c] the pre-catalyst **1** is oxidized with *m*-chloroperoxybenzoic acid (*m*CPBA) in the presence of tosylic acid (TsOH) likely giving **2**-OTs and *m*-chlorobenzoic acid (*m*CBA) based on previous DFT calculations. The reaction mixture also contains significant amounts of water, as TsOH is typically utilized in its monohydrate form. Consequently, the spectra analysis was expected to become complicated due to dynamic ligand exchange processes at the iodine(III) center involving tosylate, *m*-chlorobenzoic acid (*m*CBA) and tosylate. While the IR spectrum will be that of a mixture of all these components, the VCD will only show signals arising from constituents attached to the chiral iodane.

In order to ensure that reliable band assignments can be made and to establish the suitability of the computational approach for the prediction of IR and VCD spectra, we first carried out a VCD analysis of the iodine(I) pre-catalyst **1** in $CDCl_3$ (Figure^^1<figr1>). The experimental IR spectrum of **1** shows only few strong bands in the IR spectrum, while the VCD spectrum features many strong and characteristic bands even in the spectral regions in which the IR spectrum shows only weak and broad bands. This highlights that IR and VCD intensities are not directly correlated, and strong IR bands do not necessarily result in strong VCD bands and vice versa. The simulation of the IR and VCD spectra was based on a comprehensive conformational analysis of **1**, which was carried out by considering all rotatable bonds and systematically generating input structures for geometry optimizations at DFT level (B3LYP/def2TZVP/IEFPCM($CHCl_3$); cf. Table^^S1 for details on the optimized

conformers. While the core structure of **1** is found to possess only eight conformations, we obtained more than 120 conformers due to the conformational flexibility of the TIPS protective group. Considering the individual IR and VCD spectra of the conformers, Boltzmann-weighted spectra were generated. As can be noted from the comparison in Figure^^1<xfigr1>, the match between computed and experimental spectra is very high and even minor features are well reproduced.

After this successful benchmark based on the catalyst **1** in its stable reduced state, we continued to investigate the structure of the oxidized iodane. The required oxidation reaction (Scheme^^1<xschr1>) was carried out under standard conditions by stirring a 0.13^^M solution of the pre-catalyst **1** in $CDCl_3$ for 2^^hours at room temperature with equimolar equivalents of *m*CPBA and TsOH. The crude sample solution was subsequently transferred from the reaction vessel to the IR cuvette and the IR and VCD spectra were recorded. As anticipated, the IR spectrum of this reaction mixture showed strong signals in the carbonyl stretching region (>1700^^cm$^{<M->1}$) and in the spectral range 1300--1200^^cm$^{<M->1}$, in which S<C=>O stretching vibrations of the tosylate are expected (cf. Figure^^2<figr2>A). A clear indication that the activation reaction of the iodine(I) to an iodine(III) species has actually taken place cannot immediately be deduced. In contrast, the experimental VCD spectrum shows significant changes, especially in the range below 1330^^cm$^{<M->1}$. Here, the sign changes in the range 1310--1250^^cm$^{<M->1}$ and the changes in the range 1150--1000^^cm$^{<M->1}$ are worth highlighting. It is also important to note that the IR bands arising from the side products, such as the carbonyl stretching bands, do not feature VCD signatures, so that the experimental spectrum arises purely from the active species.

With the experimental VCD spectrum clearly showing spectral changes compared to the pure catalyst, we assumed that the iodine(III) species **2**-OTs was formed. We thus began to carry out spectra predictions for **2**-OTs hoping that the match with the experimental spectrum would be as good as in case of **1** and that the IR spectrum with all the additional bands of the side products would not be required for the analysis. In our initial report of this catalyst, we proposed conformational preferences of **2**-OTs based on DFT calculations.[9c] These previously calculated geometries were used as starting points for an even more

comprehensive search. Due to the conformational flexibility of the TIPS group, we evaluated more than 700 conformers, which were grouped into five conformer families (Figure^^3<figr3>, top; Table^^S2). The structures **2**-OTS-c1 to -c5 differ in the conformation of the chiral side group. In addition, within the pairs c1/c2 and c3/c4, the configuration of the iodine ligand sphere is inverted, that means, the OH and OTs groups are interchanged in c1/c2 while the OH changes its position from one side of the aryl ring to the other in c3/c4. Although we used a different level of theory for the DFT-calculations compared to our initial paper, we obtain the same order of conformer energies: **2**-OTs-c1, which features an OH<M.><M.><M.>N interaction with the triazole ring, is favored by 0.94^^kcal^mol<M->1 over **2**-OTs-c3, in which an I<M.><M.><M.>N interaction is present.

As demonstrated for **1**, the Boltzmann-averaged IR and VCD spectra of **2**-OTs were generated based on the single conformer spectra. A comparison with the experimental spectra, which is provided in Figure^^2<xfigr2>B, directly reveals that the match of the VCD spectra is not sufficient. This becomes especially obvious in the range of 1350--1200^^cm<M->1, in which the predicted VCD spectrum is inverted in sign compared to the experimentally observed VCD pattern. Likewise, yet anticipated, the assignment of bands in the IR spectra is difficult due to the presence of a variety of yet to be identified side products.

In an attempt to aid the assignment of the computed IR spectrum to the experimental one, we recorded the IR spectra of *m*CBA and TBA-OTs as representatives for the single component side products. While the carbonyl band region could be assigned to *m*CBA, both components' IR spectra could not explain the broad bands between 1250--1150^^cm<M->1 in the spectrum of the catalyst. Interestingly, the IR spectrum of an equimolar mixture of *m*CBA and TBA-OTs was found not to be a mere superposition of the single components' spectra (Figure^^2<xfigr2>C). Instead, the strong band centered at approx. 1200^^cm<M->1 in the spectrum of TBA-OTs splits into two broad bands at 1260 and 1160^^cm<M->1, which almost coincide with the yet unassigned bands in the catalyst spectrum. As confirmed by comparison with a computed spectrum (Figure^^S1), *m*CBA and OTs prefer to form the anionic hydrogen bonded complex [*m*CBA-OTs]<M-> and hence neither of these substrates serve as a direct ligand attached to the hypervalent iodine center. Consequently, our initial structural

assumption of **2**-OTs being present in solution with a significant coordinative interaction between the tosylate and the iodine(III) as part of the hypervalent bond is disproved.

Investigating this hypothesis in more detail, we generated cationic structures **2**$^+$-c1--**2**$^+$-c5 by removing the tosylate group from the 15 lowest energy structures of each of the five conformer families (cf. Figure^^3<xfigr3>, bottom, Table^^S3). Without the tosylate group, significant structural changes were noted in all but the **2**-OTs-c1 conformers. Most importantly, a hydrogen bond between the I<C->OH and the methoxy group could be observed which could not be formed before due to steric reasons (**2**-OTs-c2) or due to the presence of the preferential interaction with the tosylate (**2**-OTs-c3 to -c5). For **2**$^+$-c3 and **2**$^+$-c4, significant interactions between the triazole nitrogen and the hypervalent iodine atom are implied by a short N<C->I distance of 2.4^^Å. This interaction was further confirmed by an NBO analysis (see Supporting Information),[18] that underlines the coordinative nature of the triazole-iodine interaction. Even more important is the change in conformer energies: In the cationic form, the N-stabilized structures **2**$^+$-c3 and **2**$^+$-c4 become the major populated species with a more than 6^^kcal^mol$^{\text{<M->1}}$ stabilization with respect to the reference structure c1.

As the cationic form apparently possesses notably different conformational preferences than **2**-OTs, we simulated the IR and VCD spectra using the single conformer spectra and Boltzmann weights of the conformers of **2**$^+$ (cf. Figure^^3<xfigr3>, bottom). To our delight, the computed VCD spectrum of **2**$^+$ is in excellent agreement with the experimentally observed spectrum. All VCD signatures of the activated catalyst are resembled and only a minor mismatch in the exact position of the band at 1250--1230^^cm$^{\text{<M->1}}$ is observed. Likewise, the computed IR spectrum could be considered to provide a much better match with the experiment. When including the computed IR spectrum of the anionic *m*CBA-OTs complex in the comparison, the visual agreement becomes strikingly better (cf. Figure^^S2).

In conclusion, our comprehensive VCD analysis of the activated iodine(III) catalyst **2**-OTs and its pre-catalyst **1** provides a convincing case for VCD spectroscopy being a powerful tool for the characterization of yet unknown active species in asymmetric hypervalent iodine catalysis. To the best of our knowledge, this is the first spectroscopic identification of a chiral

cationic iodosyl species (**2**$^{+}$), that is stabilized by a tethered N-heterocycle, in solution phase. The simultaneous observation of the non-coordinating anionic dimer **[*m*CBA-OTs]**<M-> in $CDCl_3$ solution has broader implications as it further supports the proposed existence of cationic ligand-stabilized iodosyl species. This study is a prime example for the capabilities of VCD spectroscopy in verifying calculated structures of in situ generated reactive asymmetric catalysts, in particular in hypervalent iodine chemistry and will improve their further rational design for specific needs.

Acknowledgements

The authors gratefully acknowledge funding by the Deutsche Forschungsgemeinschaft (DFG, German Research Foundation) under Germany’s Excellence Strategy (EXC-2033; project no. 390677874), through a research grant (ME 4267/3-1; project no. 389178573), the DFG’s Heisenberg programme (ME 4267/5-1; project no. 418661145) and the DFG’s refugee program (extension to NA955/3-1; project no. 406058799).

Conflict of Interest

The authors declare no conflict of interest.

Data Availability Statement

The data that support the findings of this study are available in the Supporting Information of this article.

<lit1><lit_a><book>V.^^V. Zhdankin, *Hypervalent Iodine Chemistry: Preparation, Structure and Synthetic Applications of Polyvalent Iodine Compounds*, Wiley, Hoboken, **2014**</book>; <lit_b><book>T. Wirth, *Hypervalent Iodine Chemistry*, Springer, Cham, **2016**</book>; <lit_c><jnl>H.^^Zhang, T. Wirth, *Chin. J. Org. Chem.* **2021**, *41*, 65--70</jnl>; <lit_d><book>K. Ishihara, K. Muñiz, *Iodine Catalysis in Organic Synthesis*, Wiley-VCH, Weinheim, **2022**</book>.

<lit2><lit_a><jnl>A. Dahiya, A.^^K. Sahoo, N. Chakraborty, B. Das, B.^^K. Patel, *Org. Biomol. Chem.* **2022**, *20*, 2005--2027</jnl>; <lit_b><jnl>R. Kumar, F.^^V. Singh, N. Takenaga, T. Dohi, *Chem. Asian J.* **2022**, *17*, e202101115</jnl>; <lit_c><jnl>A. Bauer, N. Maulide, *Chem. Sci.* **2021**, *12*, 853--864</jnl>; <lit_d><jnl>C. Mairhofer, L.

Stockhammer, M. Waser, *Arkivoc* **2021**, *2021*, 112--127</jnl>; <lit_e><jnl>A. Flores, E. Cots, J. Bergès, K. Muñiz, *Adv. Synth. Catal.* **2019**, *361*, 2--25</jnl>; <lit_f><jnl>A. Parra, *Chem. Rev.* **2019**, *119*, 12033--12088</jnl>; <lit_g><jnl>A. Claraz, G. Masson, *Org. Biomol. Chem.* **2018**, *16*, 5386--5402</jnl>; <lit_h><jnl>S. Ghosh, S. Pradhan, I. Chatterjee, *Beilstein J. Org. Chem.* **2018**, *14*, 1244--1262</jnl>; <lit_i><jnl>X. Li, P. Chen, G. Liu, *Beilstein J. Org. Chem.* **2018**, *14*, 1813--1825</jnl>; <lit_j><jnl>E.^^A. Merritt, B. Olofsson, *Synthesis* **2011**, 517--538</jnl>.

<lit3><jnl>U.^^H. Hirt, B. Spingler, T. Wirth, *J. Org. Chem.* **1998**, *63*, 7674--7679</jnl>.

<lit4><jnl>U.^^H. Hirt, M.^^F.^^H. Schuster, A.^^N. French, O.^^G. Wiest, T. Wirth, *Eur. J. Org. Chem.* **2001**, 1569--1579</jnl>.

<lit5><lit_a><jnl>D. Macikenas, E. Skrzypczak-Jankun, J.^^D. Protasiewicz, *Angew. Chem. Int. Ed.* **2000**, *39*, 2007--2010; *Angew. Chem.* **2000**, *112*, 2063--2066</jnl>; <lit_b><jnl>D. Macikenas, E. Skrzypczak-Jankun, J.^^D. Protasiewicz, *J. Am. Chem. Soc.* **1999**, *121*, 7164--7165</jnl>; <lit_c><jnl>A.-A. Guilbault, C.^^Y. Legault, *ACS Catal.* **2012**, *2*, 219--222</jnl>; <lit_d><jnl>A. Yoshimura, S.^^C. Klasen, M.^^T. Shea, K.^^C. Nguyen, G.^^T. Rohde, A. Saito, P.^^S. Postnikov, M.^^S. Yusubov, V.^^N. Nemykin, V.^^V. Zhdankin, *Chem. Eur. J.* **2017**, *23*, 691--695</jnl>; <lit_e><jnl>A. Boelke, E. Lork, B.^^J. Nachtsheim, *Chem. Eur. J.* **2018**, *24*, 18653--18657</jnl>.

<lit6><lit_a><jnl>M. Uyanik, T. Yasui, K. Ishihara, *Angew. Chem. Int. Ed.* **2010**, *49*, 2175--2177; *Angew. Chem.* **2010**, *122*, 2221--2223</jnl>; <lit_b><jnl>M. Uyanik, T. Yasui, K. Ishihara, *Tetrahedron* **2010**, *66*, 5841--5851</jnl>; <lit_c><jnl>M. Uyanik, S. Ishizaki, K. Ishihara, *Org. Synth.* **2021**, *98*, 1--27</jnl>.

<lit7><jnl>T. Hokamp, T. Wirth, *J. Org. Chem.* **2019**, *84*, 8674--8682</jnl>.

<lit8><lit_a><jnl>S. Beaulieu, C.^^Y. Legault, *Chem. Eur. J.* **2015**, *21*, 11206--11211</jnl>; <lit_b><jnl>K. Kraszewski, I. Tomczyk, A. Drabinska, K. Bienkowski, R. Solarska, M. Kalek, *Chem. Eur. J.* **2020**, *26*, 11584--11592</jnl>; <lit_c><jnl>H. Zheng, Y. Sang,

K.^^N. Houk, X.-S. Xue, J.-P. Cheng, *J. Am. Chem. Soc.* **2019**, *141*, 16046--16056</jnl>.

<lit9><lit_a><jnl>A.^^H. Abazid, B.^^J. Nachtsheim, *Chem. Commun.* **2021**, *57*, 8822--8825</jnl>; <lit_b><jnl>A.^^H. Abazid, T.-N. Hollwedel, B.^^J. Nachtsheim, *Org. Lett.* **2021**, *23*, 5076--5080</jnl>; <lit_c><jnl>A.^^H. Abazid, B.^^J. Nachtsheim, *Angew. Chem. Int. Ed.* **2020**, *59*, 1479--1484; *Angew. Chem.* **2020**, *132*, 1495--1500</jnl>; <lit_d><jnl>A.^^H. Abazid, N. Clamor, B.^^J. Nachtsheim, *ACS Catal.* **2020**, *10*, 8042--8048</jnl>.

<lit10><lit_a><book>L.^^A. Nafie, *Vibrational Optical Actvity*, Wiley, Hoboken, **2011**</book>; <lit_b><book>P.^^J. Stephens, F.^^J. Devlin, J.^^R. Cheeseman, *VCD Spectroscopy for Organic Chemists*, CRC, Boca Raton, **2012**</book>; <lit_c>"Vibrational Optical Activity: From Small Chiral Molecules to Protein Pharmaceuticals and Beyond": <book>L.^^A. Nafie, *Frontiers and Advances in Molecular Spectroscopy*, Elsevier, Amsterdam, **2018**, pp.^^421--469</book>, <other><url>https://doi.org/10.1016/B978-0-12-811220-5.00014-9</url></other>.

<lit11><lit_a><jnl>J.^^M. Batista,^^Jr., E.^^W. Blanch, V. da^^Silva^^Bolzani, *Nat. Prod. Rep.* **2015**, *32*, 1280--1302</jnl>; <lit_b><jnl>C. Merten, T.^^P. Golub, N.^^M. Kreienborg, *J. Org. Chem.* **2019**, *84*, 8797--8814</jnl>; <lit_c><jnl>P.^^L. Polavarapu, E. Santoro, *Nat. Prod. Rep.* **2020**, *37*, 1661--1699</jnl>.

<lit12><jnl>C. Merten, *Eur. J. Org. Chem.* **2020**, 5892--5900</jnl>.

<lit13><lit_a><jnl>M. Mahlau, B. List, *Angew. Chem. Int. Ed.* **2013**, *52*, 518--533; *Angew. Chem.* **2013**, *125*, 540--556</jnl>; <lit_b><jnl>S. Liao, B. List, *Angew. Chem. Int. Ed.* **2010**, *49*, 628--631; *Angew. Chem.* **2010**, *122*, 638--641</jnl>.

<lit14><jnl>C. Merten, C.^^H. Pollok, S. Liao, B. List, *Angew. Chem. Int. Ed.* **2015**, *54*, 8841--8845; *Angew. Chem.* **2015**, *127*, 8967--8971</jnl>.

<lit15><lit_a><jnl>N.^^M. Kreienborg, C. Merten, *Chem. Eur. J.* **2018**, *24*, 17948--17954</jnl>; <lit_b><jnl>N.^^M. Kreienborg, C.^^H. Pollok, C. Merten, *Chem. Eur. J.* **2016**, *22*, 12455--12463</jnl>.

<lit16><jnl>T.^^P. Golub, C. Merten, *Chem. Eur. J.* **2020**, *26*, 2349--2353</jnl>.

<lit17><jnl>T.^^P. Golub, C. Merten, *Phys. Chem. Chem. Phys.* **2021**, *23*, 25162--25169</jnl>.

<lit18><other>*NBO 7*, E.^^D. Glendening, J.^^K. Badenhoop, A.^^E. Reed, J.^^E. Carpenter, J.^^A. Bohmann, C.^^M. Morales, P. Karafiloglou, C.^^R. Landis, F. Weinhold, Theoretical Chemistry Institute, University of Wisconsin, Madison, **2018**</other>.

Figure^^1 Experimental and computed IR and VCD spectra of the chiral iodoarene pre-catalyst **1**. The molar absorptivity ε and differential molar absorptivity Δ*ε* are given in units of M<M->1^cm<M->1. Experimental conditions: 0.24^^M, 100^^µm optical pathlength, $CDCl_3$. Computational details: B3LYP/def2TZVP/IEFPCM($CHCl_3$), 6^^cm<M->1 HWHH, frequency scaling factor of 0.98.

Figure^^2 A)^^Comparison of experimental IR and VCD spectra of the activated catalyst (respectively the reaction mixture) **2**-OTs and the pre-catalyst **1**; Experimental conditions for **2**-OTs: 0.13^^M, 100^^µm optical pathlength, $CDCl_3$. B)^^Overlap of the computed spectra of **2**-OTs and the cationic form **2**$^+$ with the experimental data; Computational details: B3LYP/def2TZVP/IEFPCM($CHCl_3$), 6^^cm<M->1 HWHH, frequency scaling factor of 0.98. C)^^Experimental IR spectra of *m*CPBA, TBA-OTs, *m*CBA and an equimolar mixture of *m*CBA and TBA-OTs. The molar absorptivity *ε* and differential molar absorptivity Δ*ε* are given in units of M<M->1^cm<M->1.

Figure^^3 Lowest energy representatives of the five conformer families of **2**-OTs (top) and its cation form **2**$^+$ (bottom) and the relative energies (in kcal^mol<M->1) as obtained at B3LYP/def2TZVP/IEFPCM($CHCl_3$) level of DFT. Conformer family c1 is set as reference structure for both sets of structures. The depict distances are those of the intramolecular hydrogen bond O<C->H<M.><M.><M.>N or O<C->H<M.><M.><M.>O and the I<M.><M.><M.>N and I<M.><M.><M.>O<C=>S interactions. The angle represents the torsional angle of the O<C->I bond with respect to the aryl ring plane. The identified non-coordinating anion for **2**$^+$, [*m*CBA-OTs]<M->, is not shown.

Scheme^^1 Representative examples for in situ generated chiral hypervalent iodine compounds.
